# Screening For Mental Health Issues Among Hospital Reception Staff

**DOI:** 10.1192/j.eurpsy.2026.11319

**Published:** 2026-07-31

**Authors:** H. Ziedi, W. Gdich, N. Chaouch, S. Belaam, S. Bey, G. Amri, R. Ghachem, N. Mechergui

**Affiliations:** 1 Occupational health department, Habib Thameur Hospital, Tunis; 2 Department B of Psychiatry, Razi Hospital, Mannouba, Tunisia

## Abstract

**Introduction:**

Hospital reception staff face constant stress as the first point of contact for patients. Their mental health needs are often overlooked despite high-pressure work environments. Screening for psychological issues is crucial to support their well-being and prevent burnout. Early identification promotes staff resilience and enhances overall hospital performance.

**Objectives:**

To assess using a validated questionnaire, the level of psychological distress among hospital reception staff.

**Methods:**

This is a descriptive cross-sectional study conducted in the occupational medicine department of Habib Thameur Hospital in Tunis, involving hospital reception staff, using the 12-item General Health Questionnaire (GHQ12). Severe psychological distress was defined as a GHQ12 score ≥20.

**Results:**

We asked 35 reception agents. The population was predominantly female (57%) with an mean age of 31± 4 years. The mean length of service was 14± 7 years. Fifty-seven per cent of participants had a university level of education. Comorbidity was noted in 48% of participants, 10% of whom had hypertension. Anxiety-depression disorder was found in 23% of respondents, and 26% reported taking anxiolytic medication. The average GHQ12 score was 28.71. Forty percent of counter staff experienced severe psychological distress. Moderate psychological distress was noted in 37% of the population.

**Conclusions:**

A significant proportion of hospital reception staff experience severe psychological distress. Regular mental health screening and targeted support are essential to prevent burnout. Early interventions can enhance staff well-being and improve overall patient care.

**Disclosure of Interest:**

None Declared

